# Efficacy of azvudine plus dexamethasone in severe hospitalized patients with Omicron infection: a prospective multicenter study

**DOI:** 10.3389/fcimb.2024.1390098

**Published:** 2024-11-22

**Authors:** Meng-Lan Zhang, Xiao-Ying Wei, Nan Su, Jung-Hong Jiang, Guo-Peng Xu, Da-Xiong Zeng

**Affiliations:** ^1^ Department of Pulmonary and Critical Care Medicine, The Fourth Affiliated Hospital of Soochow University, Suzhou Dushu Lake Hospital, Suzhou, China; ^2^ Department of Pulmonary and Critical Care Medicine, Medical Center of Soochow University, Suzhou, China; ^3^ Department of Pulmonary and Critical Care Medicine, First Affiliated Hospital of Soochow University, Suzhou, China; ^4^ Department of Pulmonary and Critical Care, The Affiliated Suzhou Hospital of Nanjing Medical University, Suzhou Municipal Hospital, Suzhou, China; ^5^ Gusu School, Nanjing Medical University, Suzhou, China

**Keywords:** COVID-19, Omicron, azvudine, dexamethasone, severe disease

## Abstract

**Background:**

Azvudine (AZV), the first Chinese oral anti-coronavirus disease 2019 (COVID-19) drug, has shown substantial clinical benefits to viral clearance and prognosis in patients with mild and common COVID-19. However, there is no evidence in severe hospitalized COVID-19 patients.

**Methods:**

In this multicenter study, we analyzed 209 severe hospitalized COVID-19 patients in four hospitals. All the clinical data and the 28-day composite outcomes were recorded. All of the patients were categorized into two groups according to drug: the dexamethasone (DXM) group and the azvudine plus dexamethasone (AZV+DXM) group.

**Results:**

There were no differences in sex, age, BMI, and underlying diseases between the two groups. The ratio of the 28-day composite outcome was lower for the AZV+DXM group than that for the DXM group (16.97% *vs*. 31.82%, *p* = 0.029). The viral clearance time was shorter in the AZV+DXM group than in the DXM group (7.32 ± 2.57 *vs*. 8.55 ± 2.34 days, *p* = 0.017). The PaO_2_/FiO_2_ levels on day 5 (258.89 ± 55.22 *vs*. 233.12 ± 60.51, *p* = 0.026) and day 10 (289.48 ± 44.09 *vs*. 261.52 ± 37.34, *p* = 0.015) were higher in the AZV+DXM group than the DXM group. However, data on the hospitalization duration of the two groups were similar. Cox analysis showed the benefit of AZV+DXM in the subgroups of ≥65 years old, multiple organ dysfunction syndrome (MODS), cerebrovascular disease, C-reactive protein (CRP) ≥70mg/L, and D-dimer ≥1 µg/L.

**Conclusion:**

This study is the first to indicate that treatment with AZV+DXM might benefit severe Omicron-infected patients compared with DXM treatment alone. This finding demonstrates, at least partly, the necessity of antiviral treatment in severe patients.

## Introduction

Coronavirus disease 2019 (COVID-19) remains a major threat to global health, especially in China, as its prevention and control measures change (Lu et al., 2023; Zheng et al., 2023). Numerous drugs have been repurposed or developed to treat patients with COVID-19, including nirmatrelvir–ritonavir, molnupiravir, and azvudine (AZV) (Zhang et al., 2021).

As the first Chinese oral anti-COVID-19 drug, AZV was suggested for the treatment of patients with COVID-19 (Yu and Chang, 2020; Chen and Tian, 2023; Zhu, 2023). Several clinical studies have demonstrated the benefit of AZV in patients with mild and common COVID-19. A pilot study showed that AZV treatment in mild and common COVID-19 could shorten the mean time of the first nucleic acid negative conversion to 2.5 days (Ren et al., 2020). Another study indicated that AZV treatment is associated with significantly lower risks of composite disease progression outcome and all-cause death in real-world clinical practice (Deng et al., 2023). Some other reports also showed the efficacy of AZV in patients with mild-to-moderate COVID-19 (Sun et al., 2023; Yang et al., 2023). A number of studies compared the efficacy and safety of AZV *vs*. Paxlovid in patients with mild-to-moderate COVID-19 with high risk factors, although the results differed (Dian et al., 2023; Gao et al., 2023; Zhao et al., 2023; Zhao et al., 2023). To date, all antiviral drugs for COVID-19 have been suggested for subjects with mild or common severity disease with high risks, including those over 60 years old and those with diabetes or cardiovascular diseases (Zhang et al., 2021). No antiviral drugs have been granted for patients with severe COVID-19, and these patients continue to suffer from severe acute respiratory syndrome coronavirus 2 (SARS-CoV2) infection.

For COVID-19 patients with severe or critical illness, glucocorticoids might inhibit cytokine storm and modulate inflammation-mediated lung injury, limiting the progression to respiratory failure and death (Snow et al., 2023). Dexamethasone (DXM) and methylprednisolone have been proven to reduce the 28-day mortality in patients with COVID-19 who are receiving either invasive mechanical ventilation or oxygen alone (Tomazini et al., 2020; Ranjbar et al., 2021; RECOVERY Collaborative Group et al., 2021). However, glucocorticoids have been demonstrated to delay the viral clearance or extend the time of nucleic acid negative conversion (Chen et al., 2020; Pantazopoulos et al., 2021; Tang et al., 2021; Lin et al., 2023). These could increase the risk of repeated viral damage and secondary cytokine storm. In these COVID-19 patients, it is unclear whether antiviral treatment will benefit those with severe COVID-19 or not.

The co-administration of antiviral agents and immunomodulation could serve as a potential strategy to aid viral clearance and to reduce the risk of genetic diversification (Koeckerling and Barker, 2021). A few studies have focused on the combination of remdesivir and DXM in COVID-19, but with different and even contrasting conclusions. Several studies have indicated that treatment with remdesivir plus DXM is associated with a significant reduction in mortality and length of hospitalization and a faster SARS-CoV2 clearance (Marrone et al., 2022; Wong et al., 2022). However, other studies found no association with shorter hospitalization or lower in-hospital mortality (Gressens et al., 2022; Sattoju et al., 2023). Furthermore, no evidence was found for the combination of other antiviral drugs with glucocorticoids in patients with COVID-19.

In this prospective multicenter study, we evaluated the safety and efficacy of azvudine plus dexamethasone (AZV+DXM) *versus* DXM alone in patients with severe COVID-19.

## Methods

### Study design

During the period from December 15, 2022, to April 30, 2023, we prospectively conducted a cohort study of hospitalized COVID-19 patients in three centers (i.e., the Fourth Affiliated Hospital of Soochow University, the First Affiliated Hospital of Soochow University, and the Affiliated Suzhou Hospital of Nanjing Medical University). All patients were diagnosed with positive RT-PCR for the Omicron variant of SARS-CoV2. All subjects were given corresponding treatments by their attending physician according to the severity of the disease and the accessibility of drugs. We only enrolled patients with severe disease treated with DXM or with AZV+DXM. The exclusion criteria were: 1) younger than 18 years; 2) received AZV therapy for less than 5 days; 3) received invasive ventilation on admission; 4) with immunodeficiency or immunosuppressive therapy; 5) pregnant or in the lactation period; and 6) with missing clinical prognosis information and other data.

The use of AZV and DXM was in accordance with the Chinese Guideline of COVID-19 diagnosis and treatment. AZV was administered orally at 5 mg/day (for patients with normal renal function) or at 3 mg/d ay (for patients with renal function failure) for 7–14 days. DXM was intravenously injected at 6–10 mg/day for 7–10 days.

Severe subjects were defined as those with one of the following: respiratory rate ≥30, or lung infiltrates >50%, or oxygen saturation ≤93%, or PaO_2_/FiO_2_ ≤300 mmHg. Critical subjects were defined as those with one of the following: shock, requiring intensive care unit management due to a combination of other organ failures, and respiratory failure requiring ventilator treatment.

This study was approved by the Institutional Review Board of the Fourth Affiliated Hospital of Soochow University (220143). Written informed consent was waived as we only collected all clinical data from anonymized data according to the policy for public health outbreak investigation of emerging infectious diseases issued by the National Health Commission of the People’s Republic of China.

### Data collection

The demographic characteristics (age and sex) and the clinical data (coexisting disorders, laboratory parameters, dosage and duration of AZV and DXM treatment, and the 28-day outcomes) of the participants during hospitalization were collected. PaO_2_/FiO_2_ was calculated based on the blood gas analysis at admission, on days 5–7, and on day 10 or discharge. All of the data were independently reviewed and entered into the computer database by two physicians. The severity of COVID-19 on admission was recorded.

The primary endpoint was the composite outcome of disease progression, including all-cause death, intensive care unit admission, and noninvasive or invasive mechanical ventilation, whichever came first. The secondary endpoints were viral clearance, PaO_2_/FiO_2_, and hospitalization time.

### Statistical analysis

SPSS (version 13.0) was used for statistical analysis. Continuous variables were presented as median and were compared using a *t*-test or the Mann–Whitney *U* test. Categorical variables were presented as *n* (%) and were compared using the chi-squared test or Fisher’s exact test. Survival curves were determined with the Kaplan–Meier method using the log-rank test. A two-tailed *p*-value <0.05 was considered statistically significant. Hazard ratios (HRs) with 95% confidence intervals (CIs) for each outcome between groups were estimated using Cox regression models. Subgroup analyses were conducted to evaluate the robustness of the estimates. For statistical tests, the level of significance was two-tailed 0.05.

## Results

### Baseline characteristics of the Omicron-infected patients in the two groups

All of the baseline characteristics are shown in **Table 1**. There were no differences between the two groups in terms of age, sex, and BMI. The underlying diseases in the two groups were similar, except for coronary heart disease (7.88% *vs*. 22.73%, *p* = 0.008). There were no differences between the two groups in terms of lymphocyte, leukocyte, and procalcitonin (PCT) levels, liver function markers, and renal function markers. The levels of blood glucose (7.99 ± 1.40 *vs*. 10.17 ± 2.01 mmol/L, *p* = 0.018), D-dimer (2.12 ± 0.29 *vs*. 3.52 ± 1.21 μg/mL, *p* = 0.006), and alanine transaminase (ALT) (28.99 ± 1.93 *vs*. 35.85 ± 6.99 IU/L, *p* = 0.037) were lower, but the level of C-reactive protein (CRP) was higher (69.81 ± 20.72 *vs*. 59.92 ± 17.57 mg/L, *p* = 0.011), in the AZV+DXM group than those in the DXM group. Although the cardiac troponin levels were similar in the two group, the N-terminal pro-B-type natriuretic peptide (NT-proBNP) level in the DXM group was significantly higher than that in the AZV+DXM group (3,527.22 *vs*. 1,298.67 pg/mL, *p* < 0.001).

**Table 1:** Baseline characteristics of Omicron infected patients in two groups.

	AZV+DXM	DXM	x^2^/t	P value
N	165	44		
Sex,n(%)				
Male	106(64.24)	25(56.82)	0.819	0.366
Female	59(35.76)	19(43.18)		
Age, years	71.75±1.05	72.00±2.38	3.797	0.053
BMI, kg/m2	23.99±1.38	23.46±1.67	0.009	0.924
Smoking, n(%)	21(12.73)	7(15.91)	0.000	1
Vaccine status, n(%)	146 (88.48)	36 (81.82)	1.372	0.214
Underlying diseases, n(%)				
Hypertension	96(58.182)	31(70.46)	1.494	0.222
Diabetes	42(25.45)	14(31.82)	0.557	0.455
Coronary heart disease	13(7.88)	10(22.73)	7.039	0.008
Chronic lung disease	20(12.12)	5(11.36)	0.027	0.871
Nephropathy	13(7.88)	1(2.27)	1.738	0.187
Tumor	29(17.58)	9(20.46)	0.215	0.643
Cerebrovascular disease	16(9.69)	3(6.82)	0.395	0.530
Laboratory findings				
Leukocytes,10^9^/L	6.82±1.52	7.65±2.37	1.281	0.259
Lymphocyte, 10^9^/L	0.70±0.03	0.66±0.07	0.326	0.569
Hemoglobin,g/L	123.09±25.61	115.86±23.35	1.977	0.161
Platelet,10^9^/L	173.51±36.33	201.58±33.38	1.207	0.273
CRP, mg/L	69.81±20.72	59.92±17.57	0.719	0.011
PCT, ng/mL	2.25±1.02	0.87±0.39	1.550	0.215
LDH, U/L	304.14±44.46	292.05±40.70	0.987	0.322
Blood glucose,mmol/L	7.99±1.40	10.17±2.01	5.803	0.018
Albumin,g/L	32.24±5.42	30.99±6.69	0.495	0.483
Direct bilirubin,umol/L	5.20±1.43	5.31±1.18	0.698	0.405
Total bilirubin,umol/L	12.90±2.77	10.09±2.50	1.658	0.201
AST,U/L	35.53±9.88	32.58±7.21	0.533	0.129
ALT,U/L	28.99±1.93	35.85±6.99	4.441	0.037
Urea nitrogen,mmol/L	12.89±5.71	14.57±6.16	1.164	0.131
Cr, µmol/L	99.38±27.72	107.85±30.56	1.215	0.119
APTT,s	33.16±5.76	33.12±6.88	1.929	0.109
D-Dimer, mg/L	2.12±0.29	3.52±1.21	7.668	0.006
Cardiac troponin,pg/mL	52.13±22.81	59.98±29.92	1.722	0.122
NT-proBNP,pg/mL	1298.70±264.98	3527.22±1362.77	14.734	<0.001
Respiratory support, n(%)				
Nasal catheter	98(59.39)	27(61.36)	0.078	0.779
Venturi mask	47(28.48)	12(27.27)	0.025	0.874
high-flow nasal oxygen	20(12.12)	5(11.36)	0.019	0.891
Treatment				
Duration of AZV therapy, days	9.16±1.28	--	--	--
Average dose of AVZ (mg/d)	4.35±0.81	--	--	--
Duration of DXM therapy	10.37±2.26	11.15±2.57	1.293	0.106
Average dose of DXM (mg/d)	8.76±2.35	9.54±2.42	1.061	0.111

AZV, Azvudine; DXM, dexamethasone; BMI, Body Mass Index; CRP, C reaction protein; PCT, procalcitonin; LDH, lactate dehydrogenase; ALT, glutamic pyruvic transaminase; AST, glutamic oxaloacetic transaminase.

The ratios of the different respiratory supports (nasal catheter or high-flow nasal oxygen) were similar in the two groups. The median duration of AZV treatment was 9.16 ± 1.28 days, with a median dose of 4.35 ± 0.81 mg/day. The duration and the average dose of DXM were similar in the two groups.

### Outcomes of the Omicron-infected patients after different treatments

**Figure 1:**
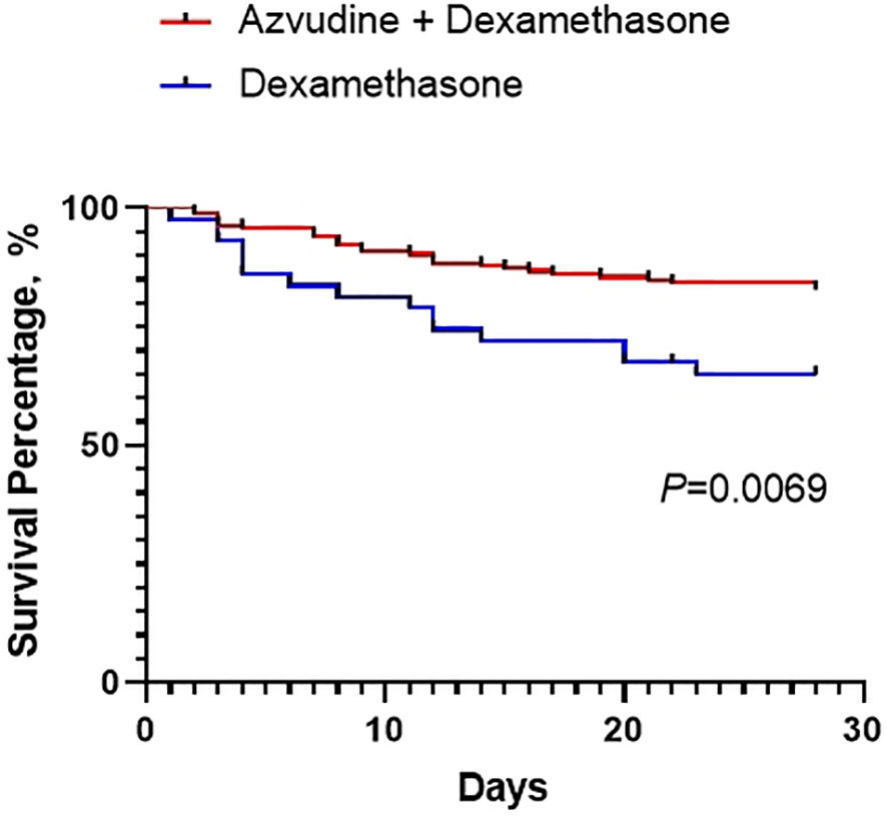
Kaplan–Meier analysis showing the 28-day composite outcomes in the two groups.

The primary and secondary outcomes are shown in **Table 2** and **Figure 1**. For the primary outcome, the 28-day composite outcome of disease progression in the AZV+DXM group was significantly lower than that in the DXM group (16.97% *vs*. 31.82%, *p* = 0.029).

**Table 2:** Outcomes of Omicron infected patients after different treatment.

	AZV + DXM	DXM	x2/t	P value
N	165	44		
28-days composite outcome	28 (16.97%)	13 (31.82%)	4.769	0.029
Viral clearance time (d)	7.32±2.57	8.55±2.34	2.467	0.017
PaO_2_ /FiO_2_				
Day 1	206.74±27.29	203.02±38.02	0.498	0.621
Day 5	258.89±55.22	233.12±60.51	2.292	0.026
Day 10 or discharge	289.48±44.09	261.52±37.34	2.508	0.015
Hospitalized time (d)	12.303±3.525	11.705±4.340	1.481	0.225

AZV, Azvudine; DXM, dexamethasone.

The median time of viral nucleic acid negative conversion was shorter in the AZV+DXM group than that in the DXM group (7.32 ± 2.57 *vs*. 8.55 ± 2.34 days, *p* = 0.017). The PaO_2_/FiO_2_ levels on day 5 (258.89 ± 55.22 *vs*. 233.12 ± 60.51, *p* = 0.026) and on day 10 (289.48 ± 44.09 *vs*. 261.52 ± 37.34, *p* = 0.015) were higher in the AZV+DXM group than in the DXM group. However, the median hospitalization times were similar in the two groups (12.31 ± 3.53 *vs*. 11.71 ± 4.34 days, *p* = 0.225).

### Changes of in the plasma parameters after treatment in the two groups

We observed some of the plasma parameters at different times in the two groups (shown in **Table 3**). About 5 days after admission, AZV+DXM treatment significantly increased the lymphocyte levels (0.63 ± 0.24 *vs*. 0.79 ± 0.25, *p* = 0.001) and decreased the CRP levels (69.81 ± 10.71 *vs*. 44.25 ± 8.85 mg/L, *p* = 0.007). This changed obviously on day 8 after admission, accompanied with a decline in the cardiac troponin levels (139.25 ± 116.94 *vs*. 79.38 ± 29.68 pg/mL, *p* < 0.001). DXM treatment decreased the CRP (58.92 ± 22.581 *vs*. 42.74 ± 21.66 mg/L, *p* = 0.003) and D-dimer levels (3.54 ± 1.16 *vs*. 2.31 ± 1.05 mg/L, *p* = 0.004) 5 days after admission, but did not improve the lymphocyte levels until the 8th day.

**Table 3:** Changes of plasma parameters after treatment in two groups.

	Azvudine + Dexamethasone (n=165)					Dexamethasone (n=44)				
	Day 2	Day 5	*P* value	Day 8	*P* value	Day 2	Day 5	*P* value	Day 8	*P* value
Lymphocyte, 10^9^/L	0.63±0.24	0.79±0.25	0.003	0.91±0.06	<0.001	0.64±0.13	0.71±0.19	0.114	1.11±0.14	0.001
CRP, mg/L	69.81±10.71	44.25±8.85	0.007	24.25±5.73	<0.001	58.92±22.58	42.74±21.66	0.003	36.05±21.68	<0.001
Troponin,pg/mL	139.25±116.94	137.39±95.20	0.953	79.38±29.68	<0.001	188.63±98.23	164.17±80.53	0.142	169.34±59.17	0.158
D-Dimer, mg/L	1.89±0.47	2.21±0.54	0.593	2.01±0.36	0.570	3.54±1.16	2.31±1.05	0.004	2.88±1.24	0.189
LDH, U/L	310.93±20.75	345.23±79.26	0.626	310.72±37.35	0.935	315.22±62.49	301.26±57.71	0.227	323.8±49.03	0.402

CRP, C reaction protein; LDH, lactate dehydrogenase.

### Independent risk factor Cox regression analysis of the subgroups

**Figure 2:**
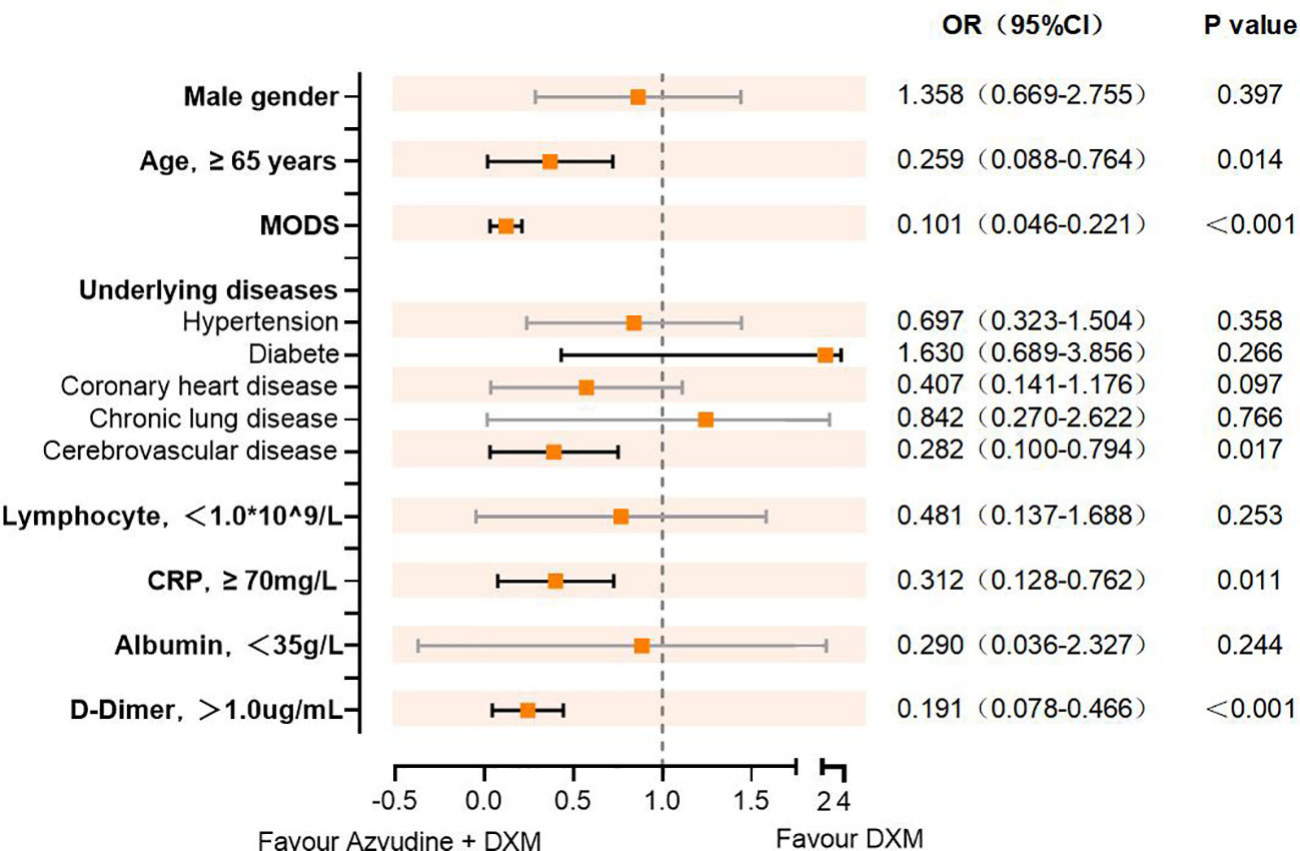
Cox regression analysis showing the independent risk factors in the different subgroups.

Subsequently, we evaluated the efficacy of AZV+DXM *versus* DXM alone in reducing the risk of composite disease progression outcome by subgroups of selected baseline characteristics. As shown in **Figure 2**, Cox analysis showed the benefit of AZV+DXM in the subgroups of ≥65 years old (HR = 0.259, 95%CI = 0.088–0.764, *p* = 0.014), MODS (HR = 0.101, 95%CI = 0.046–0.221, *p* < 0.001), cerebrovascular disease (HR = 0.282, 95%CI = 0.10–0.794, *p* = 0.017), CRP ≥70 mg/L (HR = 0.312, 95%CI = 0.128–0.762, *p* = 0.011), and D-dimer ≥1 µg/L (HR = 0.191, 95%CI = 0.078–0.466, *p* < 0.001).

## Discussion

In this multicenter prospective study, we demonstrated for the first time the efficacy of the combination of AZV and DXM in patients with severe Omicron infection. Our results indicated that the combination treatment significantly improved the 28-day prognosis of patients with severe Omicron infection. To our knowledge, this is the first study that provides evidence of the combination of antiviral drugs and glucocorticoids in patients with severe COVID-19. This study, at least partly, supports the application of antiviral drugs in patients with severe COVID-19.

Although Omicron is a mild variant with a mild pathogenicity, the severe and critical disease remains to significantly worsen the prognosis (Fan et al., 2022). Several previous reports have shown the markedly higher mortality in the elderly with Omicron infection (Wei et al., 2020; Zhong et al., 2023). RECOVERY trials and other data have provided proof that the early administration of low-dose glucocorticoids is associated with a significantly lower risk of death and decreased ventilator dependence in patients with severe COVID-19 (Tomazini et al., 2020; Ranjbar et al., 2021; RECOVERY Collaborative Group et al., 2021). However, the different types and doses of glucocorticoids might play different roles in severe COVID-19. Some reports indicated that a higher dose of glucocorticoid (DXM, 12–20 mg/day) reduced clinical worsening within 11 days after randomization compared with a low dose (Granholm et al., 2022; Taboada et al., 2022). However, more studies have demonstrated that a lower dose of glucocorticoid (DXM, 6–10 mg/day) was superior, with fewer mortality and adverse effects (Bouadma et al., 2022; RECOVERY Collaborative Group, 2023). Based on these pieces of evidence, we enrolled those subjects with severe disease treated with low- to moderate-dose DXM (6–10 mg/day). The administration of DXM significantly improved PaO_2_/FiO_2_ from day 5 and decreased CRP from day 3 after admission. These results were consistent with previous reports and demonstrated the anti-inflammatory role of low- to moderate-dose DXM in severe or critically ill patients with Omicron infection.

In this study, we excluded those subjects treated with glucocorticoids other than DXM, including methylprednisolone and hydrocortisone, for a number of reasons. Firstly, DXM was the primary glucocorticoid used in RECOVERY trials and in other studies, thus with more clinical evidence. Secondly, DXM is an inexpensive and a long-acting anti-inflammatory drug, as well as with better feasibility in hospitals. Thirdly, different types of glucocorticoids could potentially affect the efficacy of combination treatment. Although methylprednisolone is a more commonly used drug in the pulmonary department, it is undetermined whether it is superior to DXM in patients with severe COVID-19 or not. Early studies, such as the RECOVERY trial and other trials, have selected DXM as the primary glucocorticoid, which significantly improved mortality and decreased ventilator dependence. More recent studies have used methylprednisolone as the anti-inflammatory drug, which also found similar results. A number of studies showed the similar efficacy of DXM and methylprednisolone (Dastenae et al., 2022; Al Sulaiman et al., 2023), while other studies indicated the superior efficacy of methylprednisolone (Ko et al., 2021; Ranjbar et al., 2021). Further evaluation is still needed to determine the types of glucocorticoids that are more suitable for different subgroup subjects.

On the other hand, there is dispute about whether the use of glucocorticoids delays viral clearance or not. A systematic review including 44 studies showed that the viral clearance time ranged from 10 to 29 days in the corticosteroid group and from 8 to 24 days in the standard-of-care group (van Paassen et al., 2020). Multivariate Cox regression analysis also revealed that receiving systemic glucocorticoid therapy was the independent factor associated with prolonged viral shedding (Feng et al., 2020). However, some reports indicated that SARS-CoV2 clearance is not associated with glucocorticoid use (Ji et al., 2020; Spagnuolo et al., 2020). Overall, a high dose of glucocorticoids might delay viral clearance in patients with COVID-19, rather in those taking low doses of glucocorticoids (Li et al., 2021). Our previous report showed a prolonged viral RNA shedding duration of more than 45 days in COVID-19 (Zhang et al., 2022). In this study, the median time of the nucleic acid negative conversion was 8.55 days in severe Omicron-infected subjects with DXM treatment, which was longer than that in some previous reports. In our opinion, antiviral treatment might benefit shortening the viral RNA shedding duration, which theoretically ameliorates virus-associated dysfunction and cytokine storm in severe patients. However, there are a few pieces of evidence confirming the efficacy of antiviral drugs and glucocorticoids in patients with severe COVID-19.

To date, most of the clinical evidence of antiviral drugs targeting SARS-CoV2 originated from data on patients with COVID-19 of mild or common severity with high risks. There are no trials or clinical data on antiviral treatments confirmed in severe or critically ill COVID-19 subjects. This report provided direct evidence of the efficacy of antiviral drugs plus DXM in Omicron-infected patients with severe or critical illness. The combination of AZV and DXM significantly improved the 28-day prognosis compared with the DXM therapy group. Moreover, the administration of the AZV and DXM combination could elevate PaO_2_/FiO_2_ and the lymphocyte count earlier (from day 5 and day 3, respectively). The median time of nucleic acid negative conversion was also shorter in the AZV+DXM group than in the DXM group. This result was consistent with those of previous studies on remdesivir plus DXM in patients with COVID-19 requiring supplemental O_2_ therapy (Wong et al., 2022; Bernal et al., 2023; Leding et al., 2023). In these reports, remdesivir plus DXM treatment was associated with a significant reduction in mortality and length of hospitalization and a faster SARS-CoV2 clearance compared with DXM alone. However, some reports have identified that the combination of remdesivir with DXM did not bring any additional benefits (Gressens et al., 2022; Sattoju et al., 2023). These contrasting results might be due to a number of factors. Firstly, the administration times of remdesivir and DXM were different. Remdesivir was given prior to or after DXM or the two drugs were co-initiated simultaneously in different trials. These could have affected the results. Secondly, the characteristics of the enrolled subjects were markedly different, including the percentages of moderately ill and severe or critically ill subjects, the race, ages, and sex. Thirdly, most of the studies were retrospective, and the dose (6–20 mg/day), duration (7–14 days), and route (oral or intravenous) of DXM therapy were vastly different. Therefore, a prospective or a randomized controlled trial with a standard project of combination treatment would be more convincing.

Our study and the previous report provided evidence for two points. Firstly, antiviral treatment should not only be suggested in COVID-19 patients with high risk factors but also be granted in severe or critical COVID-19 patients, especially in patients without viral clearance. Anti-inflammatory therapy, such as glucocorticoids and anti-IL6 treatment, has been proven in severe patients. However, antiviral drugs should not be neglected in these subjects, especially in those with prolonged or higher-dose glucocorticoid treatment, which increases the risk of viral clearance delay. Secondly, the combination of antiviral treatment and glucocorticoids might benefit the prognosis of severe COVID-19 patients. Glucocorticoids inhibited the virus-associated cytokine storm and reduced organ damage. Antiviral treatment could accelerate the viral clearance and decrease the risk of viral clearance delay-associated secondary inflammation and cytokine storm. However, it is unclear whether synergies occur between antiviral drugs and glucocorticoids or not. Furthermore, it is uncertain whether different antiviral drugs (Paxlovid, molnupiravir, or AZV) combined with different glucocorticoids (DXM or methylprednisolone) might play similar roles or not. All of these questions need more well-designed and large-scale trials in order to provide evidence.

This study has some limitations. Firstly, it is a prospective observational study with a limited number of subjects. Randomized controlled trials and a larger number of subjects could provide more persuasive evidence. Secondly, we only focused on AZV+DXM in severe patients with Omicron infection. The efficacy of other antiviral drugs and other glucocorticoids remains unclear. Lastly, the number of patients in the AZV+DXM group (165 cases) was much higher than that of the DXM group (44 cases). This difference might have resulted in some statistical biases.

In conclusion, this multicenter prospective study revealed that the combination of AZV and DXM improved the 28-day prognosis in severe Omicron-infected patients. This might provide some evidence on the treatment strategy using an antiviral plus an anti-inflammatory in patients with severe COVID-19.

## Data Availability

The raw data supporting the conclusions of this article will be made available by the authors, without undue reservation.
